# Study on the Tribological Behaviour of Nanolubricants during Micro Rolling of Copper Foils

**DOI:** 10.3390/ma15072600

**Published:** 2022-04-01

**Authors:** Linan Ma, Jingwei Zhao, Mingya Zhang, Zhengyi Jiang, Cunlong Zhou, Xiaoguang Ma

**Affiliations:** 1 School of Mechanical Engineering, Taiyuan University of Science and Technology, Taiyuan 030024, China; xm966@uowmail.edu.au; 2 College of Mechanical and Vehicle Engineering, Taiyuan University of Technology, Taiyuan 030024, China; 3 Engineering Research Center of Advanced Metal Composites Forming Technology and Equipment, Ministry of Education, Taiyuan 030024, China; 4 School of Metallurgical Engineering, Anhui University of Technology, Maanshan 243002, China; ahutzmh@163.com; 5 School of Mechanical, Materials, Biomedical and Mechatronic Engineering, University of Wollongong, Wollongong, NSW 2522, Australia; jiang@uow.edu.au; 6 Shanxi Provincial Key Laboratory on Metallurgical Device Design and Theory, Taiyuan University of Science and Technology, Taiyuan 030024, China; zcunlong@tyust.edu.cn

**Keywords:** copper foils, micro rolling, nanolubricants, surface quality, tribological behaviour

## Abstract

Water-based lubricants with different fractions of TiO_2_ nanoparticles ranging from 1.0 to 9.0 wt.% were utilized to study the lubrication mechanisms during micro rolling tests and the tribological behaviour of nanolubricants during the micro rolling of copper foils. The results indicate that the application of TiO_2_ nanolubricants remarkably improves the surface quality of rolled copper foils during rolling processes. For lubricants with inadequate TiO_2_ nanoparticles, it is found that few TiO_2_ nanoparticles enter the contact regions between the rolls and foils, causing insufficient lubrication during rolling processes. Instead, for lubricants with excessive TiO_2_ nanoparticles, obvious agglomeration occurs at the contact regions and promotes the generation of voids on the surface of the rolled foils, thereby deteriorating the surface quality of the rolled copper foils. In addition, it is found that the surface quality of rolled foils is improved by utilizing a large reduction ratio. Overall, the fraction of 3.0 wt.% TiO_2_ nanolubricants is optimal to improve the lubrication conditions at the contact regions, thereby improving the surface quality of the rolled copper foils.

## 1. Introduction

Over the past few decades, microforming technology has been widely utilized in micro electromechanical systems, biomedicine, the automotive and aerospace sectors, and in other fields. A number of micro components, such as printed circuit boards, micro nozzles, micro molds, chemical micro reactors, tooth implantation, and high-tech medical devices are produced with the help of micro forming technology. Thus, research into micro forming technology is critical to promote the development of the industrial field. Meanwhile, the corresponding micro rolling technology has also developed rapidly and become a means for preparing ultra-thin strips in micro forming. It has attracted much attention due to its high efficiency forming, high precision products and high adaptability production process. Notably, lubrication critically affects yield, machine tool durability, energy consumption and surface finish during forming processes [1]. Thus, it is necessary to investigate the correlation between micro rolling and lubrication mechanisms.

With the advent of nanotechnology, nanoparticles are commonly applied to the field of tribology and lubrication. It is known that nanoparticles have good anti-wear and antifriction effects and can be used as additives for new lubricants [2]. According to the different properties and action mechanisms of additives, nanolubricant additives can be divided into mesh, tubular and spherical shapes, among which the typical types are metal, metal or non-metal oxides, sulfides, graphene and its derivatives, and composite nanoparticles. Nanoparticles can exhibit good anti-friction and anti-wear properties through specific lubrication mechanisms. At present, there are four widely recognized mechanisms of nanoparticles as lubricating particles—rolling effects, protective film, and the mending and polishing effects [3,4]. On the basis of this theory, scholars have carried out studies involving the tribological behaviour of nanolubricants. Xia et al. [5] studied the effect of nano-TiO_2_ additives in oil-in-water lubricant by contact angle and scratch test. The results show that the nano-TiO_2_ distributes throughout the water and improves the surface excess of the water in the lubricant. The exploration of the wettability of aqueous rolling nanolubricants was carried out by Kong et al. [6]. The obtained results showed that nano-TiO_2_ can alter the wettability of the aqueous rolling liquid, and the increased viscosity of nanolubricants is also beneficial to improve the lubricity of aqueous rolling liquids. Meng et al. [7] used triethanolamine to adjust the pH of graphene oxide lubricant, and found that the variation of physical characteristics enhanced the lubrication effect, and the alkaline lubricant exhibited the best lubrication performance. Xiong et al. [8] evaluated the tribological behaviour of water-based lubricant containing nano-CaWO_4_:Eu^3+^ during hot rolling. They found that CaWO_4_:Eu^3+^-pH = 10 has a high load-carrying capacity, wear-resistance, and friction-reducing properties in water-based lubricants. Wu et al. [9] studied the lubrication effects of water-based lubricants containing TiO_2_ nanoparticles and found that the water-based lubricant benefits the reduction of friction coefficient and the improvement of wear resistance of the chrome steel during rolling processes. Borda et al. [10] prepared oil-dispersible Cu nanoparticles, and found that both the lubrication conditions and the antiwear characteristics were improved owing to the nano-additive in oil. Xie et al. [11] investigated the effects of the base lubricant, with and without nanoparticles, on the tribological behaviour of magnesium alloy/steel contacts. The results showed that the added nanoparticles contribute to the improvement of the tribological properties of the lubricant. He et al. [12] synthesized MoS_2_-Al_2_O_3_ nanocomposites and found that MoS_2_-Al_2_O_3_ nanofluid exhibited outstanding effects on the improvement of tribological behaviour.

With the deepening of the study of the physicochemical and tribological behaviour of nanolubricants, scholars have also carried out a lot of research on the application of nanolubricants in rolling. Sun et al. [13] suggested that the main lubrication mechanism of MoO_3_ nanofluid is the formation of lubricating film on friction surfaces. Du et al. [14] synthesized anatase TiO_2_ modified GO (GO-TiO_2_), and found that the rolled strip lubricated by 0.5 wt.% of GO-TiO_2_ nanofluid had few defects and minimal roughness. Bao et al. [15,16] performed a series of tests to study the effect of nano-SiO_2_ on surface qualities, and experiments showed that surface qualities are improved owing to micro-rolling, polishing, and the self-repairing of nano-SiO_2_ on strip surfaces. Meng et al. [17] studied the effect of nano-TiO_2_ lubricating fluid on the surface and metallographic structure of hot rolled SS41 steel strips. It was found that refined tribological behaviour can be achieved by the nano-TiO_2_ lubricating fluid. Xia et al. [18] developed oil-in-water-based nanolubricants containing TiO_2_ nanoparticles and found that the lubrication effects can be attributed to the nanoparticles that entered the deform zone. Wu et al. [19,20] performed a series of hot rolling tests of microalloyed steel and low-alloy steel under different lubrication conditions, and found that an optimal fraction of 4.0 wt.% TiO_2_ nanoparticles demonstrates the best lubrication performance. Zhu et al. [21,22] dispersed nano-TiO_2_ in water to investigate the effects of nanoparticles on the tribological properties steel during rolling processes, and found that nano-TiO_2_ exhibits good tribological behaviour during hot rolling. Huo et al. [23,24] systematically studied surface roughness variation during micro flexible rolling processes. The results reveal that nanolubricants effectively decrease rolling traces.

The above literature review indicates that the research into the tribological behaviour of nanolubricants has been extensively conducted. Nevertheless, it is still short with regard to the investigation of the role of TiO_2_ nanoparticles during micro rolling processes. The objective of the present work is to study the tribological behaviour of TiO_2_ nanolubricants during the micro rolling processes of copper foils. In this manuscript, the effects of the fraction of TiO_2_ nanoparticles during micro rolling processes with different reductions was systematically investigated through a series of tests, elucidating the lubrication mechanisms during the micro rolling of foils.

## 2. Experimental

### 2.1. Material

A 99% pure copper foil with the dimensions of 150 × 5 × 0.2 mm^3^ was used in this study. SDBS (Sodium dodecyl benzene sulfonate) is a commonly used anionic surfactant with good surface activity and strong hydrophilicity, and PAAS (Polyacrylic acid sodium salt) was used as a thickening agent in the nanolubricant in the present work.

### 2.2. Preparation

**Figure 1:**
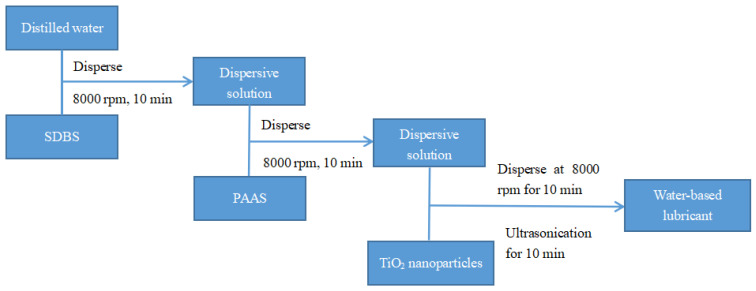
The chart of preparation of TiO_2_ nano-additive water-based lubricants.

The TiO_2_ nano-additive water-based lubricants were prepared, as outlined in Figure 1. First, SDBS was uniformly dissolved in distilled water using a disperser at 8000 rpm for 10 min. The PAAS was then added to the solution followed by the disperser, and the TiO_2_ nanoparticles were dispersed into the solution. Afterwards, the suspension was processed by ultra-sonication for 10 min in order to break down the remaining agglomeration. As a result, the TiO_2_ nano-additive water-based lubricants with good stability were prepared for further study.

The chemical compositions of the as-prepared lubricants are listed in Table 1. Different lubrication conditions (shown as numbed 1–6 in Table 1) were applied to the rolling tests. The water-based lubricants contain different mass fractions of TiO_2_ nanoparticles (from 1.0 to 9.0 wt.%) and corresponding mass fractions of SDBS. The concentration of PAAS was fixed for each type of lubricant.

### 2.3. Micro Rolling Tests

The micro rolling tests were performed on a 4-high precision laboratory micro rolling mill. The rolling tests were conducted at a rolling speed of 1 m/min and reductions of 10%, 30% and 50% under different lubrication conditions were realized, as listed in Table 1.

Various lubricants were uniformly sprayed onto the rolls prior to rolling process, and the rolls were cleaned with alcohol before the tests. The test values from three pieces of specimens were averaged in order to reduce the error generated during experiments. Notably, the capacity of absorption of various lubricants on the roll surface is inconsistent due to their different wettability.

### 2.4. Characterization and Analytical Approaches

After rolling processes, the copper foils were cleaned by alcohol. The sample surface morphologies of copper were observed under a KEYENCE VK-X1000 3D Laser Scanning Microscope (Keyence Corporation, Okasa, Japan), from which the surface roughness of the copper foils was obtained. The size of the areas of measurement was 3.7 × 4.8 mm^2^.

Furthermore, a JEOL-IT500 scanning electron microscope (SEM) (JEOL, Tokyo, Japan) equipped with an energy dispersive spectroscopy (EDS) detector (Oxford Ltd, Oxford, UK) was used to obtain additional information on the elements′ distribution and to further characterize the lubrication mechanisms.

## 3. Results

### 3.1. The Rolling Performance of Foils under Different Lubrication Conditions

**Figure 2:**
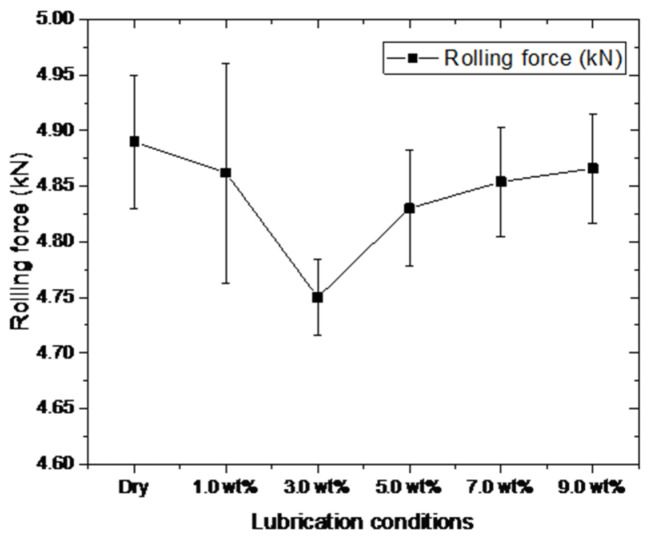
The rolling forces of copper foils during micro rolling experiments with different lubrication conditions (50% reduction).

Figure 2 shows the rolling forces obtained from micro rolling experiments under different lubrication conditions. Evidently, the highest rolling force is obtained under the dry condition, and the rolling force is gradually decreased with the application of nanolubricants. With the fraction of TiO_2_ nanoparticles increasing from 1.0 wt.% to 3.0 wt.%, drastic falling of the rolling force is observed from 4.86 kN to 4.75 kN, indicating that the lubricants with TiO_2_ nanoparticles contribute to the reduction of energy consumption induced by friction and thereby the reduction of rolling forces. Nevertheless, it is seen that the rolling forces rebounded and increased from 4.75 kN to 4.87 kN, while the fraction of TiO_2_ nanoparticles further increased from 3.0 wt.% to 9.0 wt.%. This phenomenon can be attributed to the aggregation of excessive nanoparticles that concentrates at contact regions during rolling processes [18]. The aggregation of TiO_2_ nanoparticles leads to aggravated rubbing and thereby gives a rise to the COF (Friction Coefficient), causing more energy consumption induced by friction and thus enhancing the rolling force. Additionally, the stability of the lubricating film can be hindered by the excessive nanoparticles [25], giving a rise to the COF and thereby the rolling force [26].

**Figure 3:**
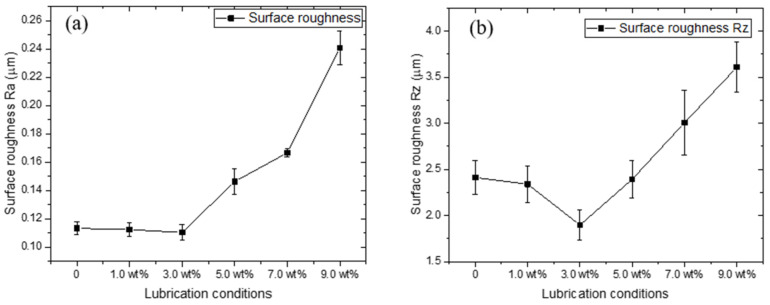
The surface roughness of the rolled copper foils under different lubrication conditions (50% reduction): (**a**) Ra and (**b**) Rz.

**Figure 4:**
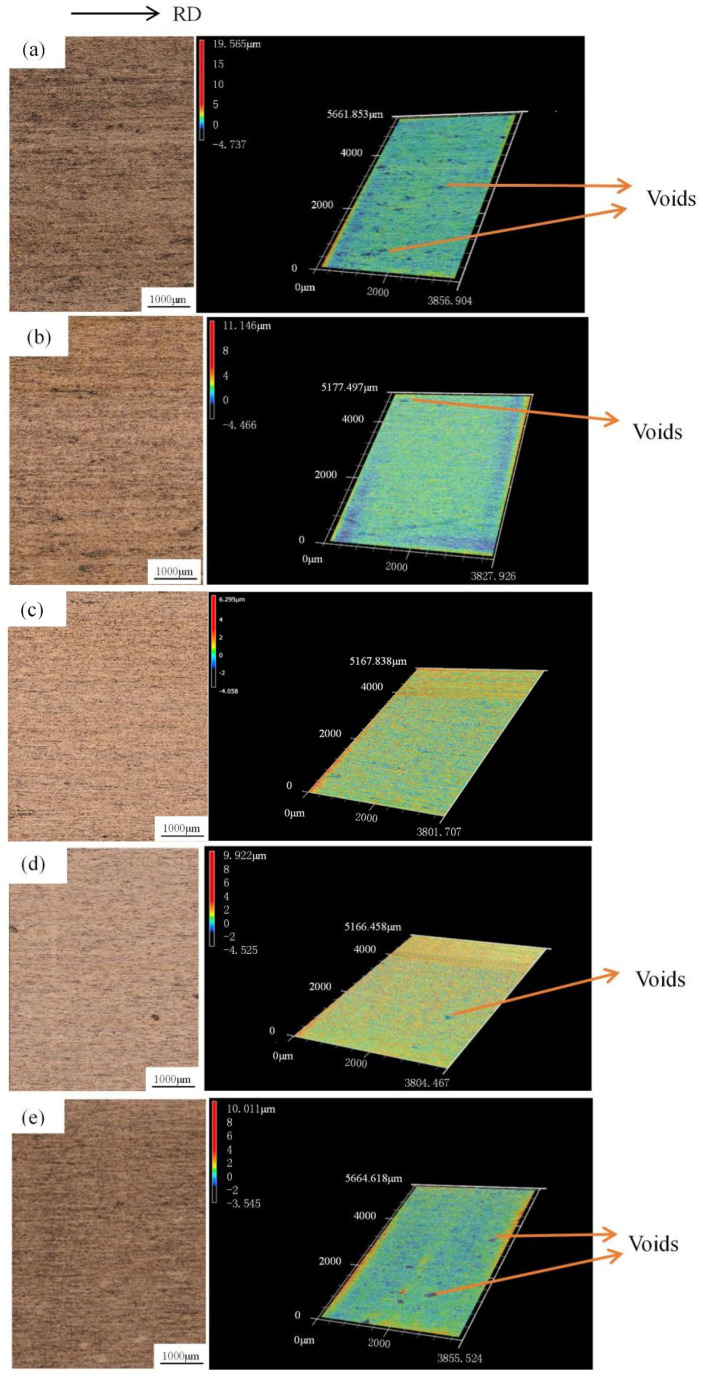

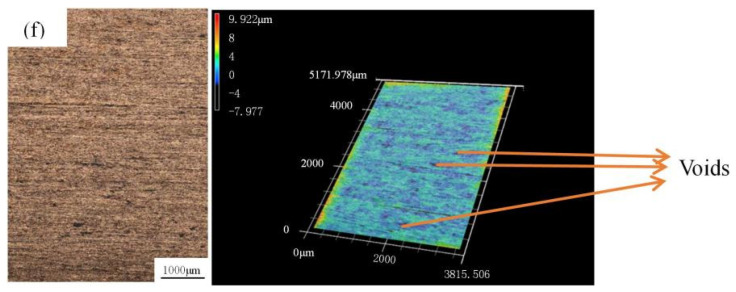
3D surface topographies of rolled copper foils (50% reduction) under (**a**) dry conditions, (**b**) 1.0 wt.%, (**c**) 3.0 wt.%, (**d**) 5.0 wt.%, (**e**) 7.0 wt.% and (**f**) 9.0 wt.%.

Figure 3 shows the variation in surface roughness of the copper foils after rolling processes. Noticeably, the lowest surface roughness value of copper foils was achieved using 3% TiO_2_ nano-additive water-based lubricants. The surface roughness values are significantly high for specimens without lubrication. With the increase of the proportion of the TiO_2_ nano-additive water-based lubricants from 0 to 3.0 wt.%, the surface roughness slightly decreased owing to improvements in lubrication conditions. In addition, compared to the Ra values, the Rz values exhibited a marked variation, indicating that the TiO_2_ nano-additive water-based lubricants successfully polished the peaks and filled in the valleys located on the surface of copper foils, thereby remarkably reducing the Rz value (the maximum distance between the peaks and valleys). In addition, it is seen that the surface roughness of copper foils gradually increased along with the proportion of lubricants further increased from 3.0 wt.% to 9.0 wt.%, which can be attributed to the smaller real contact region induced by particle aggregation [23]. The detailed 3D surface topography of the rolled copper foils were measured using a 3D laser scanning microscope, as shown in Figure 4. Noticeably, fewer and smaller voids are presented on the surfaces of copper foils rolled with 3.0 wt.% TiO_2_ nano-additive water-based lubricants, indicating that the application of TiO_2_ nano-additive water-based lubricants benefits the surface quality improvement of copper foils during micro rolling processes.

### 3.2. The Rolling Performance of Foils under Different Rolling Conditions

**Figure 5:**
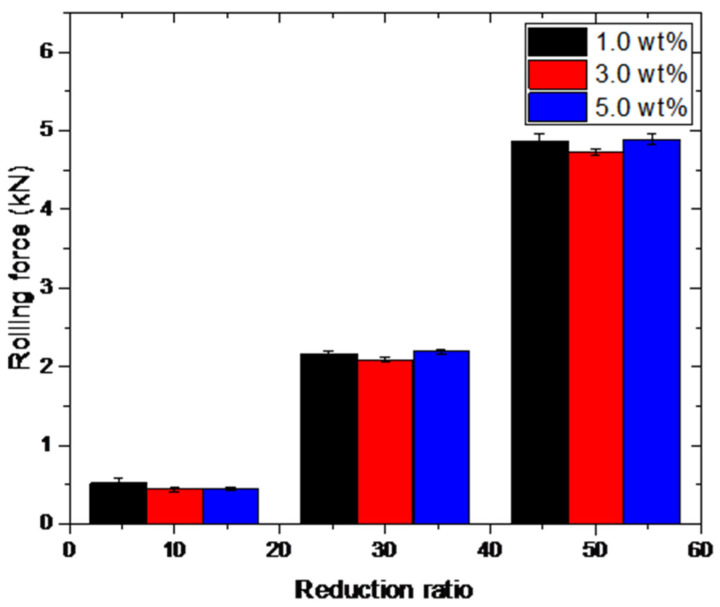
The rolling forces of the rolled copper foils under different reduction ratios.

**Figure 6:**
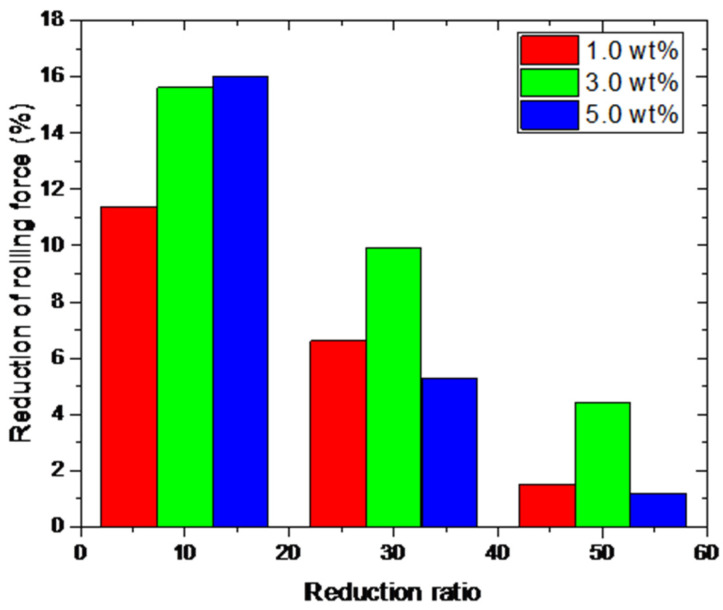
Reduction in the rolling forces of the rolled copper foils under different reduction ratios.

Figure 5 shows the rolling force obtained from micro rolling experiments under different reduction ratios ranging from 10% to 50%. In comparison with lubricants, the rolling force is significantly affected by the reduction ratio during rolling processes. For lubricants with different fractions of TiO_2_ nanoparticles, the variation in rolling force dependent on the reduction ratio exhibits a similar trend, which means that the rolling process is affected by lubrication conditions regardless of the reduction ratios. The lubrication effects of different reduction ratios on rolled copper foils are compared in Figure 6. The reduction in rolling force decreases with the increase in the reduction ratio. Comparing the results shown in Figure 4, the lubricants with TiO_2_ nanoparticles exhibit a prominent improvement in surface quality refinement while weaker effect on the reduction in rolling force corresponds to large reduction ratios.

**Figure 7:**
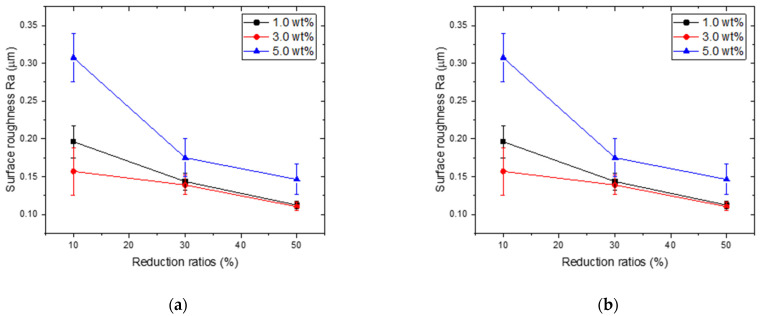
The surface roughness of the rolled copper foils with different reduction ratios during rolling processes: (**a**) Ra and (**b**) Rz.

**Figure 8:**
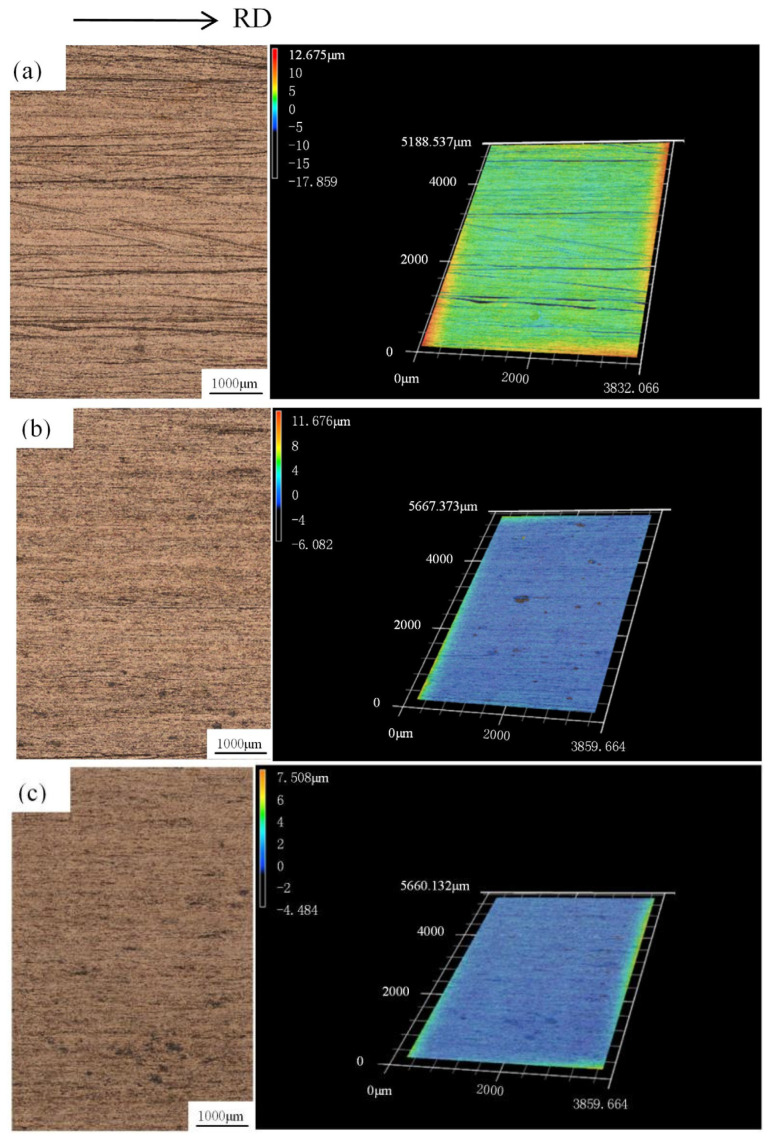
3D surface topographies of rolled copper foils with different reductions: (**a**) 10%, (**b**) 30%, and (**c**) 50%.

Figure 7 shows the variation in surface roughness of rolled copper foils under different reduction ratios. With the increase of reduction ratios from 10% to 50%, the surface roughness gradually decreased, indicating that a refined surface quality can be achieved with increased reduction ratios during rolling processes. To further evaluate the surface quality of rolled copper foils, the detailed 3D surface topography of the rolled copper foils (7.0 wt.% lubricant) are characterized using a 3D laser scanning microscope, as shown in Figure 8. For foils rolled with 10% reduction, marked rolling indentations are observed on the surface of rolled products. With the reduction ratio increases from 10% to 30%, the rolling indentation disappeared and was replaced by remarkable voids. A flat surface with few voids can be obtained under a 50% reduction, as shown in Figure 8c. The results indicate that a high reduction ratio benefits the surface quality of copper foils during rolling processes.

## 4. Discussion

### 4.1. The Effect of Lubrication Conditions

**Figure 9:**
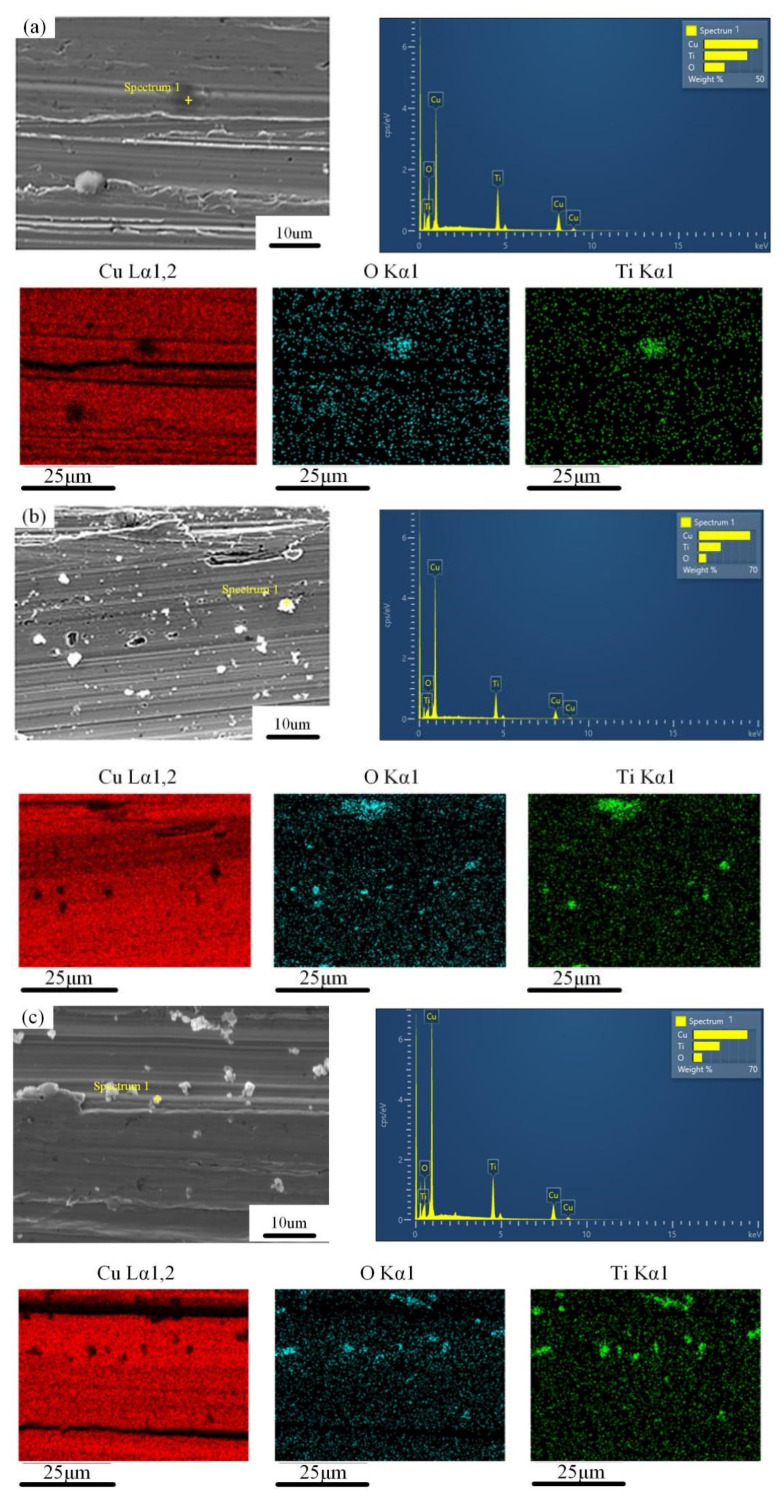

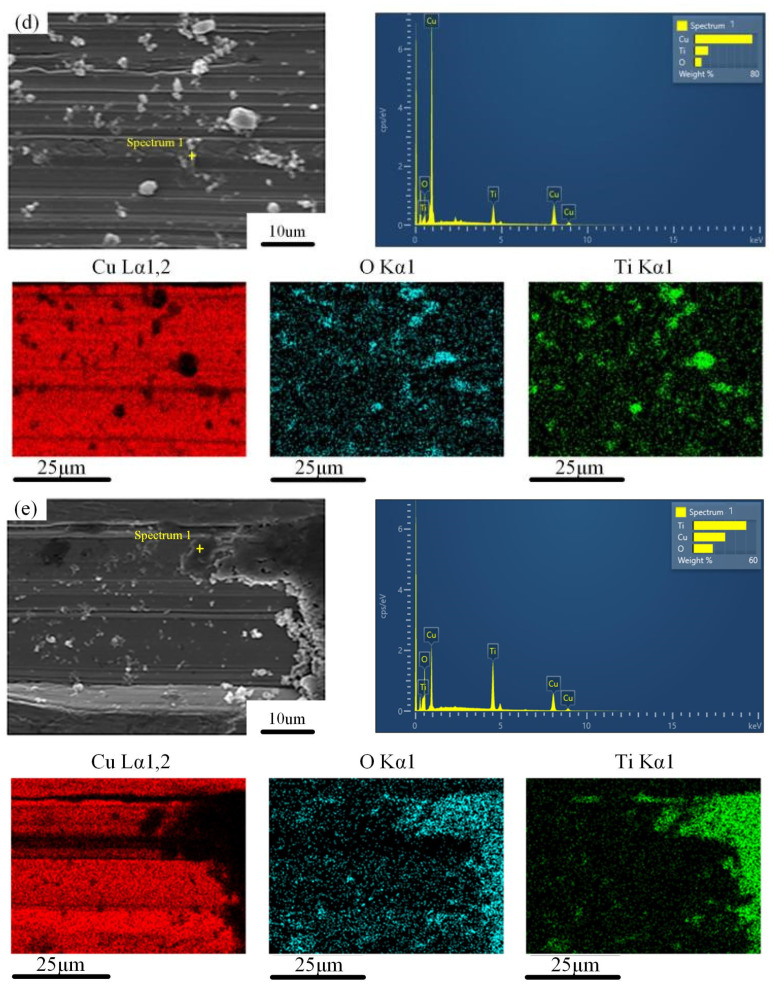
The SEM and EDS mappings of the rolled copper foils with different lubrication conditions: (**a**) 1.0 wt.%, (**b**) 3.0 wt.%, (**c**) 5.0 wt.%, (**d**) 7.0 wt.%, (**e**) 9.0 wt.%.

Figure 9 shows the SEM and EDS mappings of the rolled copper foils under different lubrication conditions. For specimens with 1.0 wt.% of TiO_2_ nanoparticles, few nanoparticles were observed on the surface of the rolled foils. The amount of nanoparticles at the real contact regions are insufficient for improving the surface quality of the rolled foils. With the proportion of lubricants increased from 1.0 wt.% to 3.0 wt.%, a large amount of TiO_2_ nanoparticles are observed and most nanoparticles are well dispersed with few agglomeration and rolling traces on the surface. Nevertheless, with the proportion of lubricants further increased from 3.0 wt.% to 9.0 wt.%, remarkable growth in the size of nanoparticles is observed on the surfaces of rolled foils, indicating that the aggregation of nanoparticles occurred and thereby affecting the surface quality of the rolled foils during rolling processes. Consequently, an optimal proportion of 3.0 wt.% of TiO_2_ nanoparticles is obtained for improving the lubrication effect of lubricants during rolling processes.

In addition, it is known that an ultra-thin film with the thickness of several hundred nanometers is formed during the rolling processes. For lubricants with 1.0 wt.% TiO_2_ nanoparticles, the mass fraction of nanoparticles is so small that they cannot form a continuous film between the rolls and foils during the rolling processes, inducing limited lubrication effects. With the fraction increases to 3.0 wt.%, a continuous film is formed during the rolling processes, reducing the friction coefficient and thereby improving the surface quality of the rolled copper foils. With the fraction further increased to 5.0 wt.%, excessive nanoparticles cannot enter the contact region, and are thereby agglomerated between rolls and foils, giving a rise to the COF and deteriorating the surface quality of rolled foils. Thus, an optimal fraction of 3.0 wt.% TiO_2_ nanoparticles is obtained to improve the lubrication effects of water-based lubricants during the micro rolling of copper foils.

### 4.2. The Effects of Rolling Conditions

**Figure 10:**
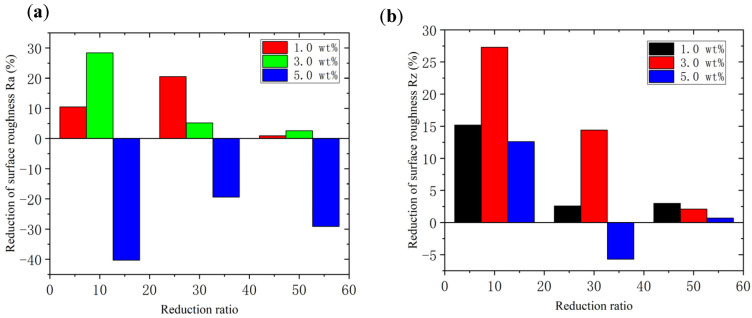
Reduction in the surface roughness of the rolled copper foils under different reduction ratios: (**a**) Ra and (**b**) Rz.

The lubrication effects of TiO_2_ nanoparticles during rolling processes are compared in Figure 10. A marked improvement in surface quality can be obtained using both 1.0 wt.% and 3.0 wt.% TiO_2_ nanoparticles. Nevertheless, for lubricants with 5.0 wt.% TiO_2_ nanoparticles, a remarkable increase in Ra is observed with few fluctuations of Rz, indicating that excessive nanoparticles cannot contribute to the improvement in lubrication effects during rolling processes. In contrast, the agglomeration of nanoparticles results in the generation of voids on the surface of foils (as shown in Figure 4), leading to remarkable undulations on the surface and thereby deteriorating the surface quality of the foils during the rolling processes. In addition, it was found that for water-based lubricants containing 3.0 wt.% TiO_2_ nanoparticles, the reduction in surface roughness (both Ra and Rz) decreased along with an increase in the reduction ratio. This phenomenon can be attributed to the improvement in surface quality following the increase in the reduction ratio. Given that the rolling force increases with an increase in the reduction ratio, the undulations on the surface of the copper foils were flattened by the large rolling force, making it difficult to further improve the surface quality by optimization of the lubrication conditions. Thus, a high reduction ratio is considered to be effective for enhancing the surface quality of rolled copper foils.

### 4.3. Lubrication Mechanisms

**Figure 11:**
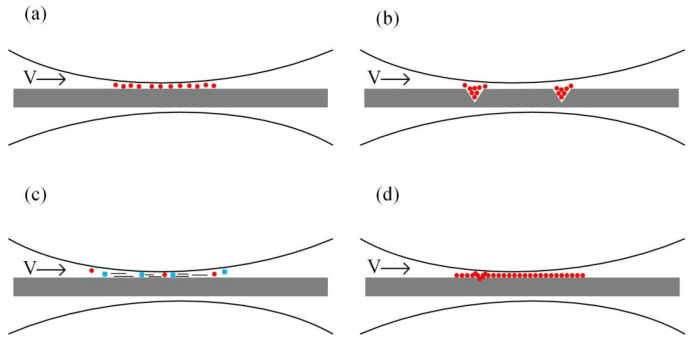
The schematic diagrams of the lubrication mechanism of TiO_2_ nano-additive water-based lubricants: (**a**) rolling effect, (**b**) mending effect, (**c**) polishing effect and (**d**) protective film.

**Figure 12:**
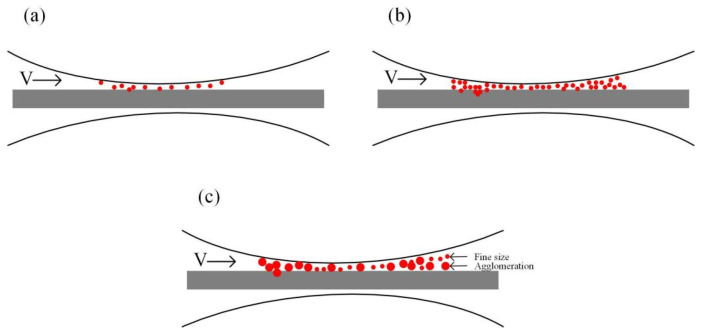
The lubrication mechanisms of TiO_2_ nano-additive water-based lubricants with different fractions of nanoparticles: (**a**) 1.0 wt.%, (**b**) 3.0 wt.%, and (**c**) 5.0 wt.%.

Numerous lubrication mechanisms have been proposed in order to demonstrate the lubrication effects of nanolubricants, including the rolling effect [26,27,28], the mending effect [29], the polishing effect [30,31], and the protective film [32,33,34], as illustrated in Figure 11. During micro rolling processes, the rolling effect (as shown in Figure 11a) improves the load carry capacity by switching from the sliding friction to the rolling friction [24]. Besides, the nanoparticles are supposed to enter the valleys and defects on the surface of foils during micro rolling processes. As a result, the microcracks are repaired and the surface defects are mended during micro rolling processes, as shown in Figure 11b. Considering that the copper foils are quite soft, the surface of copper foils are supposed to be polished and grounded using TiO_2_ nanoparticles with higher hardness, as illustrated in Figure 11c. Additionally, a thin oil film is supposed to be formed during micro rolling processes, preventing the contact between the foils and rolls and thereby reducing the friction coefficient between surfaces. In general, the rolling effect and protective film improves the surface quality of foils mainly by reducing the COF, while the mending and polishing effect improve the surface quality of foils through the repairing surface. To investigate the role of water-based lubricants with different fractions of nanoparticles during micro rolling processes, research was conducted and the schematic diagrams of the lubrication mechanism of lubricants with different fractions are demonstrated in Figure 12. Noticeably, few nanoparticles entered the rubbing surface when the fraction of the TiO_2_ nanoparticles was 1.0 wt.%, which led to insufficient lubrication during the rolling processes. With the fraction increased to 3.0 wt.%, as shown in Figure 12b, numerous fine-sized nanoparticles accumulated at the contact regions and formed a film between the rolls and foils, improving the lubrication conditions during rolling processes during the whole rolling processes. Nevertheless, with the fraction further increased to 5.0 wt.%, as shown in Figure 12c, a marked agglomeration of nanoparticles can be observed at the contact regions, deteriorating the lubrication conditions between the rolls and foils and giving rise to the wear during the rolling processes. During micro rolling processes, the formed film between rolls and foils is extremely thin with the thickness of merely hundreds of nanometers, and the smooth surfaces between foils and rolls makes it difficult for extra nanoparticles to enter the contact region. Consequently, the extra nanoparticles accumulate and rub against each other during micro rolling processes, giving a rise to the COF. Thus, an appropriate fraction of the TiO_2_ nanoparticles is critical to improve the lubrication conditions during rolling processes, and an optimal fraction of 3.0 wt.% is obtained in the present work with good lubrication effects, reduced rolling force and improved surface quality of the rolled foils.

## 5. Conclusions

### 5.1. Summary

In this work, a systematic study on the tribological behaviour of TiO_2_ nanolubricants during rolling processes was conducted. Several conclusions can be drawn as follows:

1. The application of TiO_2_ nanolubricants remarkably improves the surface quality of rolled copper foils during rolling processes. An optimal fraction of 3.0 wt.% TiO_2_ nanolubricants improves the lubrication conditions at the contact regions, thereby improving the surface quality of rolled copper foils.
2. When the amount of TiO_2_ nanoparticles is inadequate, few TiO_2_ nanoparticles enter the contact regions between rolls and foils, causing insufficient lubrication during rolling processes. In contrast, in lubricants with excessive TiO_2_ nanoparticles, marked agglomeration occurs at contact regions and promotes the generation of voids on the surface of rolled foils, thereby deteriorating the surface quality of the rolled copper foils.
3. A large reduction ratio improves the surface quality refinement during rolling processes. A remarkable improvement in surface quality can be achieved using TiO_2_ nanolubricants with different reductions ranging from 10% to 50%.

### 5.2. Future Scope

1. To further elucidate the lubrication mechanism of water-based lubricants in which nanoparticles are used in forming processes, it would be of great significance to establish an innovative numerical simulation model to explore the lubrication effects of lubricants during forming processes.
2. The use of different nanoparticles in the water-based lubricants can be investigated for improving the lubrication effects while reducing costs.
3. The role of TiO_2_ nanoparticles during micro forming processes is currently under-reported. Given that TiO_2_ nanoparticles exhibit excellent lubrication effects in the water-based lubricants used during micro forming processes, the processing and manufacturing of high-quality water-based lubricants with TiO_2_ nanoparticles would be a relevant research direction in the future.

## Figures and Tables

**Table 1:** Chemical compositions of lubricants.

Lubricant Type	Description
1	Dry
2	1.0 wt.% TiO_2_ + SDBS + PAAS + balance water
3	3.0 wt.% TiO_2_ + SDBS + PAAS + balance water
4	5.0 wt.% TiO_2_ + SDBS + PAAS + balance water
5	7.0 wt.% TiO_2_ + SDBS + PAAS + balance water
6	9.0 wt.% TiO_2_ + SDBS + PAAS + balance water

## Data Availability

Not applicable.
